# The effect of fluid resuscitation on the effective circulating volume in patients undergoing liver surgery: a post-hoc analysis of a randomized controlled trial

**DOI:** 10.1007/s10877-017-9990-5

**Published:** 2017-02-16

**Authors:** Jaap Jan Vos, A. F. Kalmar, H. G. D. Hendriks, J. Bakker, T. W. L. Scheeren

**Affiliations:** 1 Department of Anesthesiology, University of Groningen, University Medical Center Groningen, Hanzeplein 1, PO Box 30001, 9700RB Groningen, Netherlands; 2 Department of Anesthesia and Critical Care Medicine, Maria Middelares Hospital, Ghent, Belgium; 3 Department of Intensive Care Adults, Erasmus MC University Medical Center, Rotterdam, The Netherlands; 4 Division of Pulmonary, Allergy, and Critical Care Medicine, Department of Medicine, Columbia University Medical Center, New York, USA

**Keywords:** Hemodynamic monitoring, Fluid resuscitation, Circulating volume, Dynamic variables, Venous return, Mean systemic filling pressure, Liver surgery

## Abstract

To assess the significance of an analogue of the mean systemic filling pressure (Pmsa) and its derived variables, in providing a physiology based discrimination between responders and non-responders to fluid resuscitation during liver surgery. A post-hoc analysis of data from 30 patients undergoing major hepatic surgery was performed. Patients received 15 ml kg^−1^ fluid in 30 min. Fluid responsiveness (FR) was defined as an increase of 20% or greater in cardiac index, measured by FloTrac-Vigileo^®^. Dynamic preload variables (pulse pressure variation and stroke volume variation: PPV, SVV) were recorded additionally. Pvr, the driving pressure for venous return (=Pmsa–central venous pressure) and heart performance (E_H_; Pvr/Pmsa) were calculated according to standard formula. Pmsa increased following fluid administration in responders (n = 18; from 13 ± 3 to 17 ± 4 mmHg, p < 0.01) and in non-responders (n = 12; from 14 ± 4 to 17 ± 4 mmHg, p < 0.01). Pvr, which was lower in responders before fluid administration (6 ± 1 vs. 7 ± 1 mmHg; p = 0.02), increased after fluid administration only in responders (from 6 ± 1 to 8 ± 1 mmHg; p < 0.01). E_H_ only decreased in non-responders (from 0.56 ± 0.17 to 0.45 ± 0.12; p < 0.05). The area under the receiver operating characteristics curve of Pvr, PPV and SVV for predicting FR was 0.75, 0.73 and 0.72, respectively. Changes in Pmsa, Pvr and E_H_ reflect changes in effective circulating volume and heart performance following fluid resuscitation, providing a physiologic discrimination between responders and non-responders. Also, Pvr predicts FR equivalently compared to PPV and SVV, and might therefore aid in predicting FR in case dynamic preload variables cannot be used.

## Introduction

The basis of hemodynamic management in critically ill patients and in patients undergoing major surgery is formed by a rational titration of fluids, vasopressors and inotropes, with the ultimate goal to maintain sufficient tissue oxygen delivery [1]. In recent years, studies suggest that a so-called (early) goal-directed fluid therapy (GDFT) might reduce post-operative complications [2, 3]. A hallmark feature of most of the GDFT approaches is the assessment of fluid responsiveness (FR), i.e. to assess whether cardiac output (CO) will increase following a fluid bolus. Dynamic preload variables, such as pulse pressure variation and stroke volume variation (PPV and SVV, respectively), are currently the clinical gold standard for the prediction of FR [4–6]. Nevertheless, the use of these variables is limited by several factors [7–9] such as cardiac arrhythmias and (assisted) spontaneous breathing [10]. In addition, dynamic preload variables only provide a partial, simplified picture of the circulation and do not provide a quantitative estimation of the actual volume status. In more recent studies, the usefulness of the mean circulatory filling pressure (Pmsf) in guiding hemodynamic therapy has been investigated [11–14]. In short, Pmsf is the intravascular pressure that resides when there is no flow, i.e. after cardiac arrest has ensued [13, 15–17]. Pmsf is determined both by vascular filling and vascular tone and represents the driving pressure for the return of blood to the heart and thus for CO [18]. Subsequently, the true driving force of the circulation of blood is the pressure gradient for venous return (Pvr), i.e. the pressure difference between Pmsf and the right atrial pressure (or central venous pressure, CVP) [18]. Pmsf can be measured intermittently either during inspiratory hold maneuvers or during arm stop-flow measurement [12, 19, 20]. Also, an analogue of Pmsf (Pmsa) can be monitored continuously at the bedside [12, 13]. Here, Pmsa is calculated using an algorithm introduced by Parkin [21]. This algorithm incorporates actually measured conventional hemodynamic variables (MAP, CVP, CO) and patient characteristics (age, weight, length) in order to calculate Pmsa, and subsequently to calculate Pvr. Additionally, the Parkin algorithm allows calculating efficiency of the heart (E_H_), which is defined as Pvr/Pmsa, and yields a dimensionless variable ranging from 0 to 1. The significance of Pmsa, Pvr and E_H_ in FR compared to dynamic preload variables still have to be investigated.

Therefore, the aim of this study was to evaluate the changes in Pvr, PPV and SVV in both responders and non-responders to fluid administration. Secondly, we aimed to elucidate the changes in Pmsa, Pvr and E_H_ following fluid administration to allow a further physiologically based differentiation between fluid responders and non-responders.

## Methods

The current study is a post-hoc analysis of a randomized controlled trial performed in 30 patients undergoing major hepatic resection in whom we investigated the reliability of non-invasive measurement of hemoglobin concentration [22] and the ability of dynamic preload variables to predict FR [4], by administration of a 15 ml kg^−1^ fluid bolus directly after completion of hepatic resection. In accordance with the Helsinki declaration, the original study was approved by the local medical ethics committee (Ref: 2009/174, University Medical Centre Groningen, The Netherlands) and has been registered at clinicaltrials.gov (NCT01060683). Only ASA I—III patients who were scheduled for major hepatic resection were included after written informed consent had been obtained. Given that the current study represents a post-hoc analysis of de-identified data from a non-public database, no additional approval of the medical ethics committee or additional written informed consent was obtained.

In the original study, cardiac output-based data were obtained as part of routine clinical monitoring. Furthermore, each patient served as his/her own control.

Patients were excluded in case of intra-operatively diagnosed incurable disease, cardiac dysrhythmia or requirement of additional intravenous fluids to maintain hemodynamic stability before the fluid bolus was administered (the latter was required for the aim of the original study) [22].

### Anesthetic management

All patients received a standardized balanced general anesthesia after placement of a thoracic epidural catheter, as previously described [4, 22]. Patients were mechanically ventilated (volume-controlled mode) with tidal volumes of 8 ml kg^−1^ lean body mass, with PEEP set at 5 cmH_2_O. The dosage of continuously adminstered norepinephrine was titrated to keep MAP between 60 and 80 mmHg. During the phase of hepatic resection, all patients received 6 ml kg^−1^ hr^−1^ crystalloids (NaCl 0.9%, Baxter, Deerfield, IL, USA) as baseline infusion. After completion of hepatic (parenchymal) resection—but before surgical closure of the abdomen—patients were allocated to receive a 15 ml kg^−1^ fluid bolus of either crystalloids (n = 15) or colloids (n = 15) in a fixed time frame of 30 min.

FR was defined as an increase in CO, indexed to BSA (Cardiac Index, CI) by at least 20% after fluid administration. A higher-than-normal definition of FR was applied because of both the relatively large amount of fluid administered during the resuscitation phase and the increased discriminative ability of dynamic variables to predict FR in case of higher FR thresholds [23].

### Hemodynamic monitoring

All measurements took place during the 30 min period of fluid administration.

MAP was monitored invasively using a 20 G radial artery catheter and the pressure transducer was connected to the vital signs monitor (Philips MP70; Philips, Eindhoven, Netherlands). The FloTrac-Vigileo^®^ device (software V03.02, used in all patients) was connected to the arterial pressure transducer for continuous calculation of CO using an automated auto-calibrated analysis of the arterial pressure waveform. Additionally, this device calculates stroke volume variation (SVV) over a 20 s period using the formula: $\text{SVV}= {\text{ }}({\text{S}}{{\text{V}}_{{\text{max}}}} - {\text{S}}{{\text{V}}_{{\text{min}}}})/{\text{S}}{{\text{V}}_{{\text{mean}}}}$. PPV was calculated subsequently over the equivalent time frame as ${\text{PPV }} = {\text{ }}\left( {{\text{PP}}{{\text{V}}_{{\text{max}}}} - {\text{ PP}}{{\text{V}}_{{\text{min}}}}} \right){\text{ }}/{\text{ PP}}{{\text{V}}_{{\text{mean}}}}$, using dedicated software developed by the authors. Obvious artefacts were eliminated by visual inspection of the waveforms.

CVP was continuously recorded after cannulation of the right internal jugular vein using a 7 F triple lumen catheter. Both the arterial and central venous pressure transducers were zeroed and thereafter adjusted to the height of the right atrium.

The calculation of Pmsa is based on the Parkin algorithm [21], which follows the equation: ${\text{Pmsa}}\, = \,{\text{a}}\left( {{\text{CVP}}} \right)\, + \,{\text{b}}\left( {{\text{MAP}}} \right)\, + \,{\text{c}}\left( {{\text{CO}}} \right)$. Here, a and b are constant values (a + b = 1), without dimension and, based on an assumed veno-arterial compliance ratio of 24:1, a = 0.96 and b = 0.04. The value of c depends on age, weight and height, and resembles arteriovenous resistance. The interested reader is referred to the original publication for more detailed information [21]. Pmsa has been validated in experimental and clinical studies [12, 14].

Handling of data recording of the hemodynamic data was described previously [4]. All data were synchronized and were exported to Microsoft Excel 2010 (Microsoft, Redwood, MS, USA) for statistical analysis.

### Statistical analysis

Continuous variables were tested for normal distribution using the Kolmogorov–Smirnoff test. Normally distributed variables were tested using the paired or unpaired Student’s *t*-test. Non-normally distributed data were tested using the (paired) Mann–Whitney test or (unpaired) Kruskal–Wallis test.

Correlation between relevant variables was depicted as a scatter plot and coefficients of determination (R² values) were calculated.

The ability of dynamic preload variables and Pvr to predict FR was assessed using receiver operating characteristic (ROC) analysis. The areas under the ROC curve (AUROC’s) were compared using the DeLong methodology [24].

Statistical significance was set at p < 0.05. Data are presented as mean ± standard deviation.

## Results

A total of 30 patients (14 male, 16 female) received a fluid bolus and were included for analysis. Mean age of all patients was 57 ± 13 years, mean height and weight were 176 ± 7 cm and 80 ± 13 kg, respectively.

18/30 patients demonstrated an increase in CI > 20% and were regarded as fluid responders; consequently, 12/30 patients were regarded as non-responders.

Hemodynamic variables at baseline and after fluid administration are summarized in Table 1 both for fluid responders and non-responders.

**Table 1:** Hemodynamic variables at baseline and after fluid bolus administration

	Non-responders (n = 12)		Responders (n = 18)	
	Baseline	After	Baseline	After
Heart rate (bpm)	80 ± 14	79 ± 10	90 ± 19	89 ± 16
MAP (mmHg)	75 ± 11	71 ± 9	73 ± 11	76 ± 9
CVP (mmHg)	7 ± 4	9 ± 4^#^	6 ± 3	8 ± 5
CI (L min^−1^ m^−2^)	3.1 ± 0.8	3.2 ± 1.0	2.9 ± 0.8	4.2 ± 1.2*^,#^
PPV (%)	16 ± 9	9 ± 5^#^	23 ± 11^*^	9 ± 7^#^
SVV (%)	12 ± 6	10 ± 5^#^	17 ± 8*	7 ± 4*^,#^
Pmsa (mmHg)	14 ± 4	17 ± 4^#^	13 ± 3	17 ± 4^#^
Pvr (mmHg)	7 ± 1	7 ± 2	6 ± 1*	8 ± 1*^,#^
RVR (mmHg min^−1^ m^−2^ L^−1^)	2.3 (0.4)	2.2 (0.4)	2.5 (0.6)	2.1 (0.4)^#^
E_H_	0.56 ± 0.17	0.45 ± 0.12^#^	0.52 ± 0.11	0.49 ± 0.12
Norepinephrine dosage (µg kg^−1^ min^−1^)	0.16 ± 0.21	0.13 ± 0.15	0.13 ± 0.15	0.12 ± 0.12

Data are presented as mean ± SD

*MAP* Mean Arterial Pressure, *CVP* Central Venous Pressure, *CI* Cardiac Index, *PPV* pulse pressure variation, *SVV* stroke volume variation, *Pmsa* mean systemic filling pressure analogue, *Pvr* driving pressure for venous return, *E*_*H*_ cardiac performance

*Indicates p < 0.05 versus non-responder group

^#^Indicates p < 0.05 versus value before fluid administration

### Fluid administration and changes in Pmsa, Pvr, E_H_, and dynamic preload variables

Pmsa was comparable between responders and non-responders at baseline (13 ± 3 vs. 14 ± 4 mmHg; p = 0.28, Table 1). Pmsa increased both in responders and in non-responders to 17 ± 4 mmHg (p < 0.01 for both groups) after fluid administration.

**Fig. 1:**
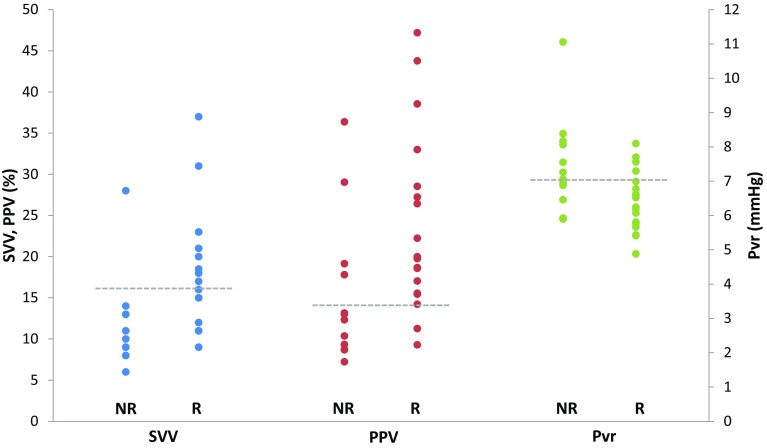
Shown are baseline individual values of SVV (*blue*), PPV (*red*) and Pvr (*green*) both for responders (R) and non-responders (NR) to fluid administration. The *dashed grey lines* represent the optimal cut-off value, as determined by ROC analysis

Pvr (= Pmsa–CVP) was lower in responders than in non-responders at baseline (6 ± 1 vs. 7 ± 1 mmHg, p = 0.02). Moreover, Pvr only increased in responders (6 ± 1 to 8 ± 1 mmHg, p < 0.01) after fluid administration. In Fig. 1, the distribution of baseline values of PPV, SVV and Pvr is shown seperately for responders and non-responders.

Heart Performance (E_H_) was comparable between responders (0.52 ± 0.11) and non-responders (0.56 ± 0.17) at baseline (p = 0.47). Importantly, E_H_ decreased following fluid administration in non-responders to 0.45 ± 0.12 (p < 0.01) but remained stable in the responders group (0.49 ± 0.12, p = 0.16). The resistance to venous return (RVR) did not differ between both groups at baseline. In responders, RVR decreased after fluid administration [from 2.5 ± 0.6 to 2.1 ± 0.4 mmHg min^−1 ^ m^−2^ L^−1 ^ (p < 0.01)], while it remained unchanged in non-responders.

Norepinephrine dosages at baseline were not significantly correlated with Pmsa, Pvr, RVR or E_H_ (all R^2^ values <0.1), which was also true after fluid administration (also, all R^2^ values <0.1).

### Prediction of fluid responsiveness (FR)

**Fig. 2:**
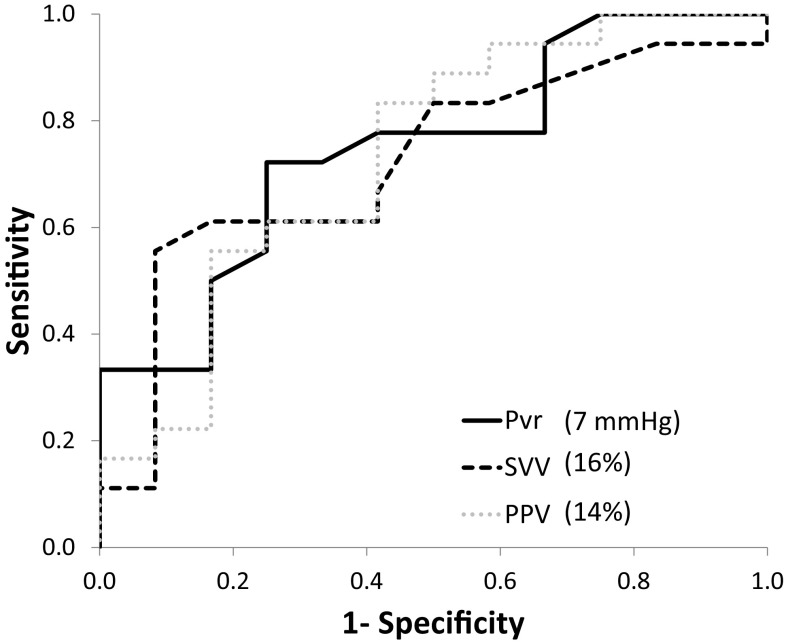
Receiver operator characteristics (ROC) Curve for assessing the prediction of FR by Pvr (*black solid line*), PPV (*grey dashed line*) and SVV (*black dashed line*). Also given are the optimal cut-off values

The AUROC of Pvr in predicting FR was 0.75 (95% Confidence Interval (CI): 0.57–0.93; p < 0.05, Fig. 2). The AUROC of PPV was 0.73 (CI: 0.54–0.92; p < 0.05), while that of SVV was 0.72 (CI: 0.53–0.91; p < 0.05), Fig. 2.

There were no significant differences between the observed AUROC’s. The optimal cut-off value for Pvr was 7 mmHg with an associated sensitivity and specificity of 72% and 75%, respectively. The optimal cut-off values for PPV and SVV were 14% and 16% respectively, with associated sensitivity and specificity values of 83/58% and 56/91%, respectively.

Of note, the AUROC of Pmsa and E_H_ to predict FR was not significant (0.48 and 0.41, respectively).

**Fig. 3:**
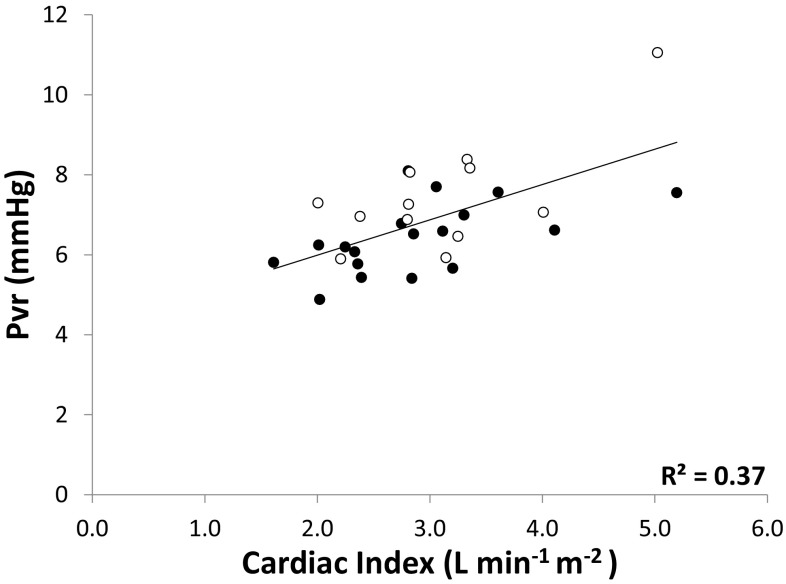
*Scatter plot* showing the correlation between cardiac index (CI, *x-axis*) and Pvr (*y-axis*) before the administration of fluid. Shown is the coefficient of correlation for all data points. (*closed circle* responders, *open circle* non-responders)

**Fig. 4:**
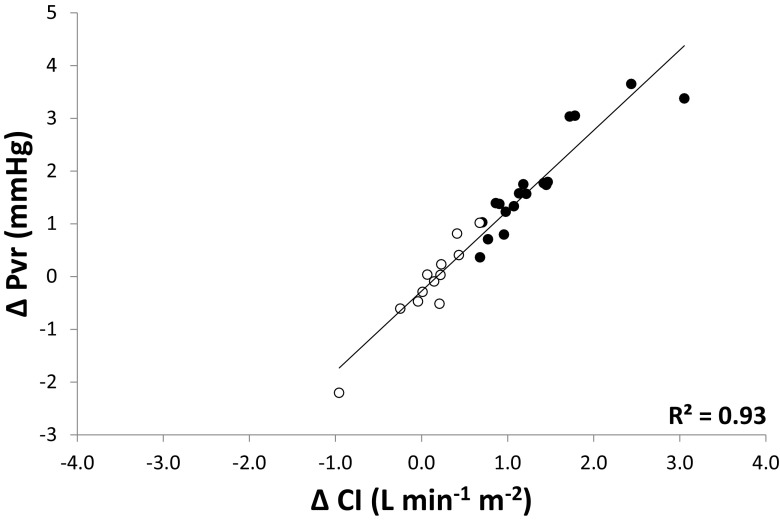
*Scatter plot* showing the correlation between the change in CI (∆CI, *x-axis*) and the change in Pvr (∆Pvr, *y-axis*) following administration of fluid. Shown is the coefficient of correlation for all data points. (*closed circle* responders, *open circle* non-responders)

There was a moderate correlation between Pvr and CI at baseline (R^2^ = 0.37; p < 0.01, Fig. 3) with no difference between responders and non-responders. Furthermore, the change in CI following fluid administration (∆CI) was strongly correlated with the corresponding change in Pvr (∆Pvr) (R^2^ = 0.93; p < 0.01; Fig. 4). Of the change in dynamic preload variables, only ∆SVV inversely correlated with ∆CI (R^2^ 0.18, p < 0.01), whereas ∆PPV did not (R^2^ 0.05, p = 0.25) (data not shown; no differences between responders and non-responders).

## Discussion

In this study, we evaluated the effects of fluid administration on the effective circulating blood volume using a cardiovascular model.

Firstly, the model-derived variables closely followed theoretically predicted volume-induced changes and allow a more detailed differentiation between fluid responders and non-responders. Pmsa increased in both groups following fluid administration. Yet, in responders, CVP did not change and as such, Pvr (Pmsa–CVP) increased which led to an increase in CI. In other words, the heart was able to generate more output from the increase in venous return. In non-responders, CVP increased to a similar extent as Pmsa and the increase in CVP helps reducing venous return.

Importantly, E_H_, decreased as the heart was unable to handle the increase in venous return, while in responders E_H_ remaine stable, i.e. the efficiency of the heart in handling an increase in venous return was maintained.

Secondly, the observation that PPV, SVV and Pvr predict FR equivalently, might suggest that Pvr can be used alternatively for the prediction of FR in case the former variables cannot be used.

### Physiologic differentiation between fluid responders and non-responders

CO is determined by the effective circulating blood volume (ECBV), the resistance to venous return and the pressure within the right atrium [25]. Pmsa, as a surrogate of ECBV, is determined both by vascular filling and tone and provides a pressure variable for the determination of flow, i.e. venous return. Subsequently, Pvr functions as the driving pressure for generating venous return and hence, CO. The current data support this theory because, according to our definition of FR (i.e. an increase in CI > 20%), Pvr increased in responders but remained unchanged in non-responders—a finding that was previously also observed in post-cardiac surgery patients receiving even a more “subtle” fluid challenge (250 ml) in comparison with our study [13]. In physiologic terms, these observations suggest that in fluid responders, the heart is able to handle the increase in Pmsa by generating more output, numerically reflected by an increase in Pvr. In non-responders, the increase in Pmsa cannot be handled by the heart. CVP increases passively, as a consequence of both increased venous return and impaired cardiac function, a mechanism described in more detail previously [26].

E_H_ (Pvr/Pmsa) reflects the Pvr—Pmsa relationship: it decreased significantly in non-responders, but remained unchanged in responders. As explained above, this observation might indicate that in these patients the heart was unable to handle the increase in Pmsa and thus, these patients did not benefit from fluid, i.e. these patients were likely to be (already) on the “flat” part of their Frank-Starling curve. We therefore speculate that this variable might be used clinically to monitor the effects of fluid administration on cardiac performance itself. Potentially, E_H_ might also demonstrate the (combined) effects of fluid administration and other factors such as inotropic medication on cardiac performance. Future studies should confirm the potential use of E_H_. Interestingly—as patients were randomized to receive either crystalloids or colloids—we found no differences in Pmsa or derived FR variables between these groups. Given the controversial differences between the intravascular effects of these fluids, this finding might be contrary to expectations, although this post-hoc analysis was not set up to study differences between fluid types, and studying these effects probably requires a larger sample size.

We observed a decrease in RVR (Pvr/CI) in responders, which is likely given that RVR will decrease if the increase in CI exceeds the increase in Pvr. This observation however contrasts previous reports in which RVR remained stable after either changes in norepinephrine dosage in septic shock patients [27] or after fluid administration in a mixed post-surgical ICU population [13]. Given our limited sample size and the complexity of our combined intervention (fluid administration, norepinephrine dosage changes), we cannot draw any (further) conclusions other than that the decrease in RVR in responders follows theoretically predicted physiologic changes.

### The assessment of fluid responsiveness (FR)

Currently, in sedated and mechanically ventilated patients, dynamic (preload) variables like PPV and SVV are established predictors of FR—both perioperatively and in the ICU—and these variables have been found superior to static variables like CVP and pulmonary capillary wedge pressure [3–5, 28–30] Like CVP, *isolated* pressure values such as Pmsa are inadequate predictors of FR, as demonstrated by an AUROC of 0.48 in our study. These static variables should therefore not be used solely [31] as a surrogate for indicating flow. Theoretically, this is also expected to apply to Pvr. Yet, We observed that FR prediction by Pvr is comparable to that of dynamic preload variables. Also, Pvr and CI were moderately correlated. These observations imply that Pvr might be used alternatively for predicting FR [32].

Interestingly, the ability to predict FR by dynamic preload variables was lower than generally reported in literature [5, 30], even though none of the recognized factors [7–9] limiting the accuracy of dynamic variables (e.g. spontaneous breathing activity, cardiac arrhytmia) was present in any of our patients. As measurements were obtained during ongoing hepatic surgery, we cannot rule out that surgical manipulation with subsequent alteration(s) of venous return, might be responsible for the decreased accuracy of dynamic variables. Also, while we found no differences between PPV, SVV and Pvr in predicting FR, the number of studied patients (n = 30) was relatively low (as reflected by relatively wide AUROC confidence intervals). It is important to reckon that results were based on *one* definition of FR (i.e. an increase of CI > 20%), while we have recently shown [23] that the ability of dynamic preload variables to predict FR is substantially dependent on the actual definition of FR: the differences in AUROC of PPV and SVV compared to our previous report [4] in the same population, serve as an example. A larger sample size would have allowed a further analysis based on multiple definitions of FR and possibly the recognition of more subtle differences in the ability of Pvr or dynamic preload variables in predicting FR.

There are no recommendations for an “optimal” Pvr value for guiding fluid management: as an example, a Pvr of 5 mmHg can result from multiple combinations of Pmsa and CVP, such as 10/5 mmHg and 20/15 mmHg, respectively. In these instances, it is questionable whether CI will increase to a similar extent. Therefore, future studies in large, perioperative patient populations or in critically ill patients should verify the assumed role of Pvr in predicting FR. Of note, a recent study in post-cardiac surgery patients suggested using the ratio between changes in Pvr and changes in Pmsa—called “volume efficiency”—as an additional predictor of FR [33].

Also, the usefulness of variables such as Pvr and E_H_, has not yet been the subject of investigation in any (early) GDFT trial. Therefore, it remains to be elucidated—as was also recently suggested [34]—whether these variables allow us to improve hemodynamics in the individual patient, together with a subsequently beneficial effect on patient outcome.

### Study limitations

Pmsf is a theoretical variable that cannot be measured clinically to assess its validity, as real-life measurement would require circulatory arrest [15]. The calculation of its analogue (Pmsa) using the Parkin algorithm [21], incorporates values of MAP, CO and CVP. An increase in CO is therefore mathematically coupled to an increase in Pmsa and hence, the definition of FR is mathematically coupled to the investigated variables Pmsa and Pvr. Ideally, Pmsf were assessed independently from CO. Pmsf can be assessed by two other methods: One of these methods uses inspiratory-hold maneuvers (requiring a sedated and ventilated patient) in which data pairs of CO and CVP are back-extrapolated to a zero CO state [19]. The other method [12] assesses Pmsf using transient stop-flow arm measurements. These two non-CO dependent methods for assessing Pmsf were regarded non-feasible in our study during ongoing surgery. Nevertheless, previously it was shown that while absolute Pmsa values may slightly differ from Pmsf values obtained using the other two methods, changes in the three Pmsf estimates were linear and “calibration” of Pmsa using a calibration factor resulted in zero bias [12]. As such, our results confirm that Pmsa can be used to track fluid-induced volume changes, yet its absolute value might be incorrect to some extent [12]. Therefore, while we used a *mathematical* approach to estimate Pmsf—which is inherently coupled to CO—it is likely that assessing Pmsf using other methods would have produced similar results. Importantly, this issue requires further research.

It has been shown previously in septic shock patients that norepinephrine titration influences venous return by exerting effects on Pmsf and resistance to venous return [27]. In our study, the flow rate of continuous norepinephrine infusion was altered to maintain MAP between 60 and 80 mmHg. Hence, Pmsa and other derived variables might have been affected by this mechanism, although we could not find any correlation between dosage(s) of norepinephrine and Pmsa or related variables. Yet, this issue deserves further elucidation in future dedicated research.

Finally, the applied algorithm incorporates hemodynamic values and combines these with demographic parameters in order to calculate Pmsa and subsequent calculation of Pvr. As the algorithm for calculation of Pmsa is based on multiple (independent) measurements (MAP, CVP and CO), the measurement error of Pmsa is an accumulation of the individual measurement errors of each variable, e.g. due to variation in the location of tip of the central venous catheter. For the latter, we verified post-hoc the correct positioning of the central venous catheter in all patients.

## Conclusions

In conclusion, bedside calculation of Pmsa, Pvr and E_H_ allow a physiology-based differentiation between responders and non-responders to fluid administration. Also, the ability of Pvr to predict FR was similar to that of PVV and SVV and therefore, Pvr might be used as an alternative method for predicting FR, especially in situations in which PPV or SVV are deemed unreliable.
